# Serological prevalence of toxoplasmosis in pregnant women in Luanda (Angola): Geospatial distribution and its association with socio-demographic and clinical-obstetric determinants

**DOI:** 10.1371/journal.pone.0241908

**Published:** 2020-11-06

**Authors:** Amélia Nkutxi Vueba, Clarissa Perez Faria, Ricardo Almendra, Paula Santana, Maria do Céu Sousa

**Affiliations:** 1 Faculty of Pharmacy, University of Coimbra (FFUC), Coimbra, Portugal; 2 Center for Neuroscience and Cell Biology (CNC), University of Coimbra, Coimbra, Portugal; 3 Centre of Studies on Geography and Spatial Planning (CEGOT), University of Coimbra, Coimbra, Portugal; 4 Centre of Studies on Geography and Spatial Planning (CEGOT), Department of Geography and Tourism, University of Coimbra, Coimbra, Portugal; Universita degli Studi di Parma, ITALY

## Abstract

We report a study on toxoplasmosis in pregnant women in Luanda, Angola, determining the seroprevalence, geospatial distribution and its association with socio-economic features, dietary habits and hygiene and health conditions. Anti-*Toxoplasma gondii* IgG and IgM were quantified in serum samples of women attended at the Lucrecia Paim Maternity Hospital between May 2016 and August 2017. The IgG avidity test and qPCR assay were used for dating the primary infection. Data were collected by questionnaire after written consent, and spatial distribution was assessed through a Kernel Density Function. The potential risk factors associated with *Toxoplasma* infection were evaluated using bivariate and multivariate binomial logistic regression analysis. Anti-*T*. *gondii* antibodies were quantified in 878 pregnant women, and 346 (39.4%) samples were IgG positive, 2 (0.2%) positive for IgM and IgG, and 530 (60.4%) negative for both immunoglobulins. The longitudinal study showed that none of the seronegative women seroconverted during the survey. Regarding other infections, 226 (25.7%) were positive for hepatitis B, while 118 (13.4%) were HIV-positive. The seroprevalence of toxoplasmosis was similar in most municipalities: 43.8% in Cazenga (28 of 64); 42.5% in Viana (88 of 207); 42.3% in Cacuaco (22 of 52); and 41.1% in Luanda ((179 of 435). In contrast, the seroprevalence in municipality of Belas was lower (25.8%; 31 of 120) and bivariate and multivariate analysis has shown a lower risk for toxoplasmosis in this area (OR 0.479, CI: 0.305–0.737; OR 0.471, CI: 0.299–0.728). The multivariate analysis has shown a significant increased risk for toxoplasmosis in women in the last trimester of pregnancy (OR 1.457, CI: 1.011–2.102), suffering spontaneous abortion (OR 1.863, CI: 1.014–3.465) and having pets at home (OR 1.658, CI: 1.212–2.269). Also, women who tested positive for hepatitis B (OR 1.375, CI: 1.008–1.874) and HIV (OR 1.833, CI: 1.233–2.730) had a significant increased risk for *T*. *gondii* infection. In conclusion, our study showed that a large number of pregnant women are not immunized for toxoplasmosis and identified the risk factors for this infection in Luanda. It is crucial to establish the diagnosis of primary maternal infection as well as the diagnosis of congenital toxoplasmosis. Our results underlined the need for diagnostic and clinical follow-up of toxoplasmosis, HIV and hepatitis B during pregnancy.

## Introduction

Toxoplasmosis is a cosmopolitan zoonosis that can affect humans as well as all warm-blooded animals, including mammals and birds. Toxoplasmosis is an infectious disease caused by *Toxoplasma gondii*, an obligate intracellular protozoan that has the ability to invade and multiply in any nucleated cell. During its life cycle, it presents three evolutionary forms: (i) the tachyzoite, (rapidly growing life stage) present during acute infection, (ii) the bradyzoite, (slow growing life stage) present during chronic infection in tissue cysts, and (iii) the sporozoite (spore-like form), protected inside an oocyst, shed by feline hosts in feces [1–3].

The most common transmission routes of toxoplasmosis are by oral means, either by eating undercooked contaminated meat that contains cysts or by ingesting water and uncooked foods contaminated with sporulated oocysts, and the congenital pathway, mother-to-child transmission during pregnancy. Less frequent transmission occurs through blood transfusion and organ transplant [1,4–6].

Congenital infection is one the most serious forms of toxoplasmosis, occurring during acute toxoplasmosis in a seronegative mother when tachyzoites present in the blood cross the placenta and infect the fetus [4,7,8]. Maternal infections are predominantly asymptomatic or cause only mild symptoms, including malaise, night sweats, myalgia, hepatosplenomegaly, swelling of the lymph nodes and fever [9]. Frequency of congenital transmission and severity of the infection varies considerably according to the gestation time at which the woman became infected. During the first trimester, transplacental transmission is relatively low (<20%), increasing up to 90% by the end of pregnancy [10,11]. Nevertheless, frequency of transmission and severity of the disease are inversely related. Congenital infection acquired in the first and second trimesters may result in severe congenital toxoplasmosis with spontaneous abortion, hydrocephaly, cerebral calcifications and mental retardation [1,8]. Whilst in late maternal infection (third trimester) the damage tends to be lower. Clinical manifestations range from chorioretinitis (occurring in 90% of cases), learning disability, sensorineural hearing loss, and cerebellar or motor dysfunction [9].

Toxoplasmosis has a cosmopolitan distribution with seroprevalence rates range from 10% to 90% [12]. The prevalence of toxoplasmosis varies dramatically between countries and often within different regions of the same country or between different communities in the same region. Generally, developing countries have a higher incidence than industrialized countries. Areas of high prevalence have been documented in Latin America, Eastern/Central Europe, the Middle East, Southeast Asia and in tropical countries in Africa. Low prevalence has been observed in North America, in South East Asia, in Northern Europe, and in Sahelian countries of Africa [12,13].

In Angola, a country in South West Africa with a population of over 25 million, few studies on toxoplasmosis have been conducted in recent years [14–16]. To date, only one study on the prevalence of antibodies to *T*. *gondii* in pregnant women has been conducted in Luanda [16] and the prevalence of congenital toxoplasmosis is not known. Also, no research to date has explored the overall seroprevalence of *T*. *gondii* infection among women in Angola, nor have the risk factors associated with the infection been examined in a regional context.

The objective of this study was to determine the seroprevalence of toxoplasmosis in pregnant women who attended a referral maternity facility located in Luanda (Angola), and to provide a detailed analysis of the geographical distribution. The study also evaluated the influence of demographic variables, socio-economic features, dietary habits, and hygiene and health conditions on the *T*. *gondii* infection. This knowledge is essential for the development of effective prevention and control strategies and the implementation of the Toxoplasmosis Surveillance Program in the Angolan National Health System network.

## Material and methods

### Ethical considerations

The present study has been approved by the Research Ethics Committee of Lucrécia Paim Maternity Hospital (LPMH) through the National Institute of Public Health of the Republic of Angola (n^o^ 301019; S1 File).

Participating individuals provided a written signed informed consent prior to sample collection and for participants younger than 18 years, informed consent was provided by parents or guardians after a detailed explanation of the objectives of the work.

### Study area

**Fig 1:**
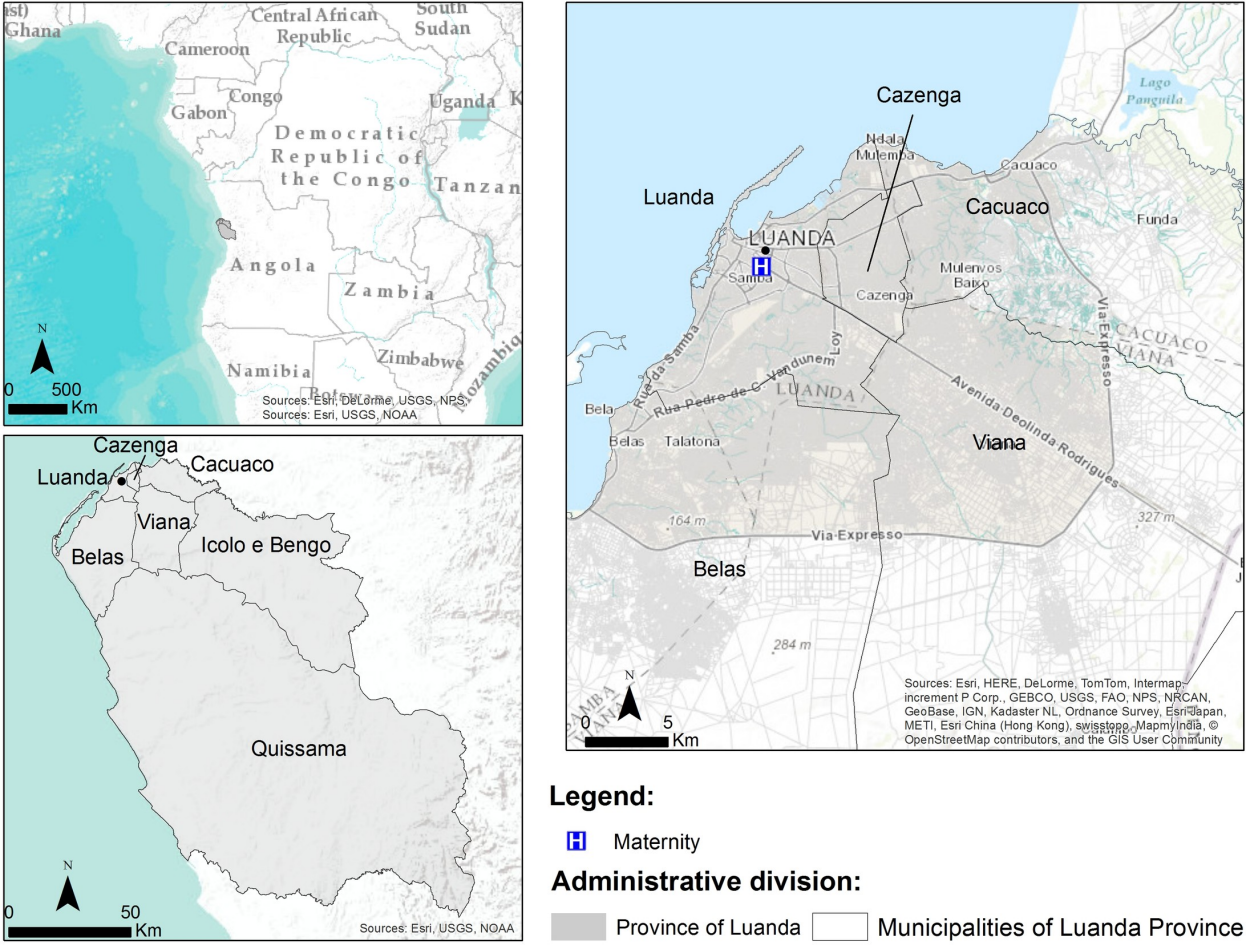
Localization of the province of Luanda, Angola, and their municipalities: Luanda, Cazenga, Cacuaco, Cazenga, Ícolo and Bengo, Luanda, Quissama and Viana.

The Republic of Angola is located on the west coast of sub-Saharan Africa (Fig 1). It is one of the largest countries of the continent, with a surface of 1,246,700km^2^ and near 25.8 million inhabitants [17]. Angola, like most developing countries, has a fairly young population [17]. The population aged 0–14 years is 12,196,496, representing 47% of the total resident population [17]. It is estimated that there are 48% of men and 52% of women and that children under 5 years old constitute 15% of the total population [17]. The economically active population is less than 50%, with a large number of state and family dependents. Women of reproductive age (15–49 years) make up about 44% and the estimated fertility rate is 6.2 children per woman [18].

Luanda is the capital and largest city in Angola, located on the coast with the Atlantic Ocean (Fig 1), is also the primary port and economic center of the country. According to the last census conducted in 2014, Luanda has a population of 6,945,386 inhabitants and is composed of 7 municipalities: Belas, Cacuaco, Cazenga, Ícolo and Bengo, Luanda, Quissama and Viana [17].

### Study population

Pregnant women monitored for routine prenatal assessment at LPMH, a reference maternity in Angola, located in Luanda, were included in the study. The referred maternity is a public health institution specializing in maternal and child health care, teaching and research. The health institution offers outpatient and inpatient services, has 400 beds for hospitalization.

The study included women aged from 15 to 47 years, who had a pregnancy confirmed by ultrasonography and laboratory tests. For the obstetric follow-up we counted on the collaboration of the medical and nursing team of the department of obstetrics of the LPMH.

### Sociodemographic, clinical, behavioral and housing characteristics of the pregnant women

An individual survey questionnaire was used to collect epidemiological variables (age, educational level, occupation, marital status and residing area), clinical information (gestational age and number of births), risk factors for *T*. *gondii* infection such as pets’ species at home, especially cats, where they defecate and the type of food consumed, frequent contact with animals other than their own, presence of rodents in or near the house, contact with soil/ gardening, if recently had a blood transfusion, if recently had some sting with needle or sharp objects and past medical history (history of miscarriage, prematurity, presence of any underlying disease as hepatitis B and HIV) (S2 File).

Information’s about basic sanitation (availability of drinking water, toilets at home and garbage disposal) and alimentary habits (breeding animals for home consumption; consumption of raw/undercooked meat, game, raw eggs, and unwashed raw/vegetables/fruit) were asked. Some questions about the knowledge of toxoplasmosis, if pre-natal consultation is performed in all pregnancies and history of abortion were also included in the questionnaire (S2 File).

### Blood sample collection and laboratory procedures

**Fig 2:**
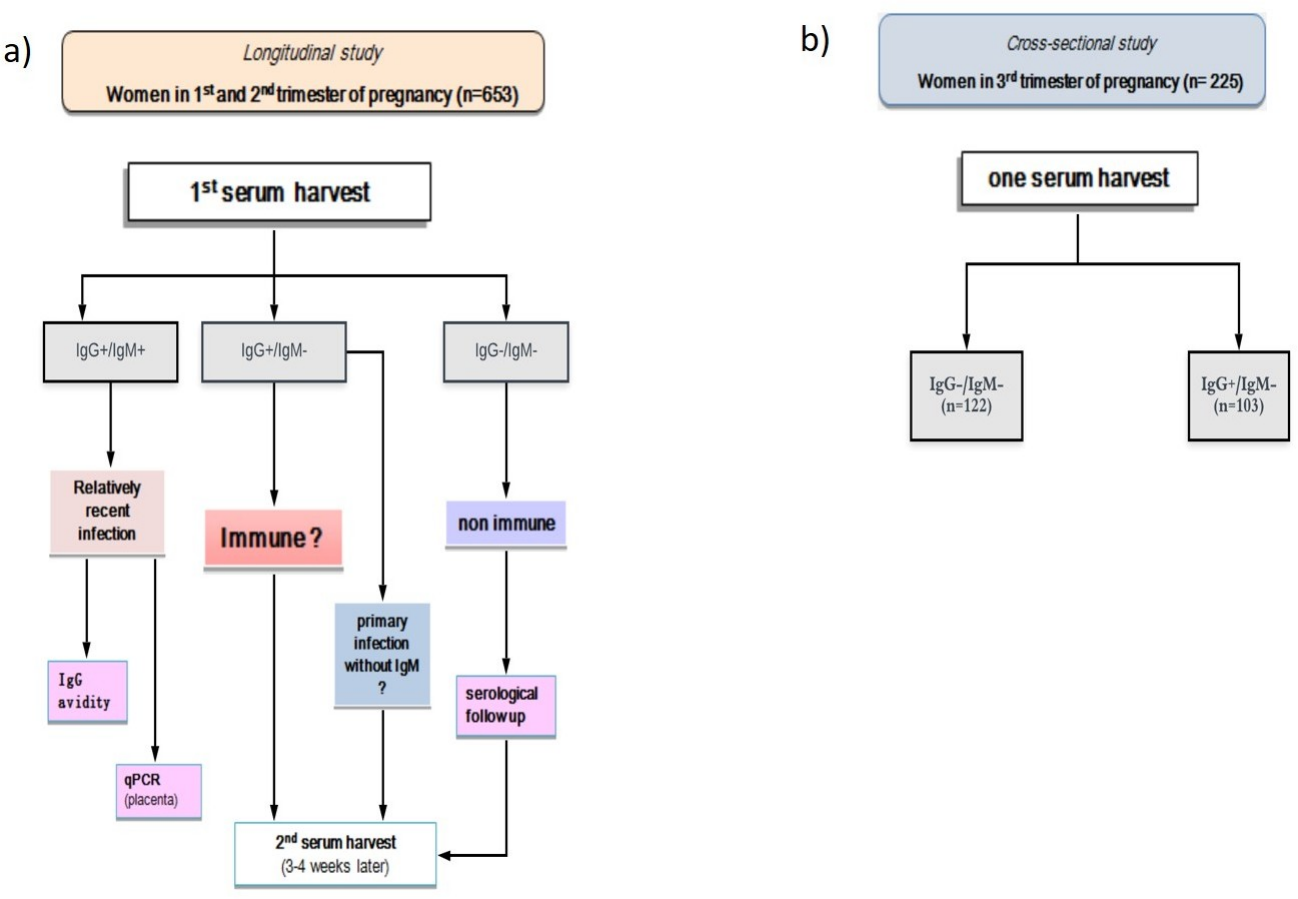
Flow chart of study sample and analyses for Toxoplasmosis survey: (a) flow chart focuses on the longitudinal study, including only women in the 1st and 2nd trimester of pregnancy; (b) flow chart focuses on the cross-sectorial study, including only women in the 3rd trimester of pregnancy.

A cross-sectional and a longitudinal survey were carried out from August 2016 to May 2017. The longitudinal study included pregnant women in the first and second trimesters of pregnancy (n = 653) and the cross-sectional study included pregnant women in the third trimester (n = 225) (Fig 2).

From each pregnant woman venous blood was collected and serum samples were obtained after centrifugation. These serum samples were immediately transferred (properly packaged in dry ice) to the Clinical Pathology Service of Clínica Sagrada Esperança (Luanda) and kept at -80°C until serological analysis.

Quantification of anti-*T*. *gondii* IgG and IgM antibodies was performed by Electrochemiluminescence (ECL) using commercially available kits for COBAS e411 (Roche Diagnostics, Germany). The results of anti-*T gondii* IgG and IgM were expressed as IU / mL and COI, respectively. Values <1 IU / mL of anti-*T gondii* IgG antibodies were considered negative and values > 3 IU / mL were considered positive. Values <0.8 COI of IgM anti-*T gondii* antibody were considered negative and values ≥1.0 COI were considered positive.

Serum follow-up samples were collected in the second and third trimesters for seronegative women included in the longitudinal study (Fig 2). All these sera were tested concurrently with the initial sample. Also a second sample was collected at least 3–4 weeks after the initial sample of all women suspected of having active primary infection by *T*. *gondii*, i.e. IgG-/IgM+. On samples that were IgG+/IgM+, a IgG avidity test was performed (Elecsys® Toxo IgG Avidity, Roche Diagnostics, Germany). Values <70% were considered as low avidity (<70 Avi% = low avidity) and values ≥80% were considered as high avidity (≥80 Avi% = high avidity.) Values 70% ≤ value <80% are considered gray zone.

Detection of anti-HIV antibodies (total anti-HIV-1, anti-HIV-2 and HIV-1 p24 antigen) was performed by Enzyme Linked Fluorescent Assay (ELFA) using commercial kits for VIDAS (HIV DUO Ultra, HIV5 and HIV P24 II) (Biomerieux, Portugal). The Hepatitis B infection was characterized by the detection of antigens (anti-HBs Ag, HBe Ag) and antibodies Anti-HBc Total II,HBc IgM, Anti-HBsT II) using ELFA commercial kits for VIDAS (Biomerieux, Portugal).

### Molecular study

In IgM and IgG seropositive women, placental samples were collected by the medical team on the day of the babies' birth and immediately transported to Clínica Sagrada Esperança. DNA was extracted from placental fragment using the NZY Tissue gDNA Isolation kit (Nzytech, Portugal) according to the manufacturer’s instructions and kept at -20°C. Extracted DNA was analyzed by nested-PCR using primers for B1 gene locus (Table 1) [19]. Amplification of the B1 gene was performed with primers B1F1 and B1R1 in the primary PCR, and with B1F2 and B1R2 primers in the secondary reaction, generating a 213bp fragment. All reactions contained 12.5μL of DyNAzymeII PCR Master Mix (Finnzymes, Finland), 1μL of each primer (10pmol/μL), 2μL of extracted DNA and 8.5μL of sterile water, performing a final volume of 25μL. PCR was carried out on the MJ Mini™ Thermal Cycler (BioRad). After an initial denaturation of 94°C for 5min, a set of 35 cycles was run, each consisting of 30s at 94°C, 30s of annealing (54°C for the primary reaction, 60°C for the second), and 60s at 72°C, followed by a final extension step of 5min at 72°C. Toxoplasma DNA sample and nuclease free distilled water were used as positive and negative controls, respectively. The PCR products were analyzed on 1.5% agarose gels stained with ethidium bromide and visualized using a gel documentation system (Uvitec, UK).

**Table 1:** List and sequence of primers for *Toxoplasma gondii* B1 gene locus.

Primer	Sequence (5´-3´)	Target
B1F1	CCGTTGGTTCCGCCTCCTTC	*T*. *gondii B1* gene (GenBank accession no. AF179871)
B1R1	GCAAAACAGCGGCAGCGTCT	
B1F2	CCGCCTCCTTCGTCCGTCGT	
B1R2	GTGGGGGCGGACCTCTCTTG	

### Geospatial analysis

The address of pregnant women was collected during the interview allowing the identification of the residence place. This information was converted into geographic coordinates (latitude and longitude) through the www.google.pt/maps/. The spatial distribution of pregnant women was assessed through a Kernel Density Function that allowed the estimation of the intensity of events across a surface (S3 File).

### Statistical analysis

The data entry was carried out using Excel software and analyzed by Statistical Package for the Social Sciences (SPSS) version 20. Percentages were used to perform exploratory analysis of the categorical variables and quantitative variables are presented as mean ± standard deviation (±SD).

The statistical analysis was developed in three steps: 1) Fisher's exact test was used to compare the number of events of seropositivity between the risk factors identified; 2) Bivariate binomial logistic regression were developed to assess the effect of different risk factors on toxoplasmosis seroprevalence; 3) Multivariate binomial logistic regression models were applied to account for the the impact of age on the effect of different risk factors on toxoplasmosis seroprevalence. The level of statistical significance was set as p<0.05, and Odds Ratio (OR) and 95% Confidence Intervals (95% CI) were computed.

## Results

Between August 2016 and May 2017, a total of 878 pregnant women from Luanda (Angola) were tested for anti-*T*. *gondii* antibodies. No clinical complaint related to disease was made at the time of the serosurvey.

The study has revealed 348 (39.6%) positive samples for anti-*T*. *gondii* antibodies (Table 2). Of these, 346 (39.4%) samples were positive for the IgG antibody and 2 (0.2%) for both IgG and IgM antibodies. In total, 530 (60.4%) of women were seronegative for *T*. *gondii* antibodies. In the two positive samples for IgM and IgG antibodies, the IgG avidity test results (high avidity) showed that these women had a chronic *Toxoplasma* infection.

**Table 2:** Distribution of IgG and/or IgM antibodies for *Toxoplasma gondii* of seropositive and seronegative pregnant women from Luanda (Angola).

Seroprevalence	Positive (%)	Negative (%)
Overall prevalence	348 (39.6)	530 (60.4)
Anti-*T*.*gondii* IgG	346 (39.4)	532 (60.6)
Anti-*T*.*gondii* IgM	0 (0)	878 (100)
Anti-*T*.*gondii* IgM/IgG	2 (0.2)	876 (99.8)

In the longitudinal study, a total of 653 pregnant women were followed during gestation: 408 (62.5%) were seronegative and of these 178 (33.6%) were in their first trimester of pregnancy and 230 (43.4%) in the second trimester (Table 3). These women were followed throughout the pregnancy and remained seronegative until the end of the pregnancy. Of the 245 (37.5%) seropositive pregnant women, 97 (27.9%) were in the first trimester of pregnancy, and 148 (42.5%) in the second trimester (Table 3). In the cross-sectional study, 225 pregnant women in the third trimester were included, with 103 seropositive for *T*. *gondii* and 122 seronegative (Table 3). Once these women were in the last trimester of gestation no more samples were collected.

**Table 3:** Characteristics of *Toxoplasma gondii* seropositive and seronegative pregnant women, according to the independent categorical variables evaluated.

Characteristics	Positive n (%)	Seroprevalence (%)	Negative n (%)	Seroprevalence (%)	Total
**Age group (years)**					
≤ 19	7(2.0)	17.5	33 (6.2)	82.5	40 (4.6)
20–25	53 (15.2)	27.5	140 (26.4)	72.2	193 (21.9)
26–35	222(63.8)	44.0	282 (53.2)	56.0	504 (57.4)
36–47	66 (19.0)	46.8	75 (14.2)	53.2	141 (16.1)
**Education**					
Illiterate	5 (1.4)	55.6	4 (0.7)	44.4	9 (1.0)
Elementary School	137 (39.4)	40.1	205 (38.7)	59.9	341 (38.9)
High school	172 (49.4)	39.3	266 (50.2)	60.7	439 (50.0)
Higher education	34 (9.8)	38.2	55 (10.4)	61.8	89 (10.1)
**Occupation**					
Homemakers	86 (24.7)	42.8	115 (21.7)	57.2	201 (22.9)
Public function	103 (29.6)	36.7	178(33.6)	63.3	281 (32.0)
Student	77 (22.1)	40.3	114 (21.5)	59.7	191(21.8)
Restoration	50 (14.4)	45.0	61(11.5)	55.0	111(12.6)
Street vendor	20 (5.7)	37.0	34 (6.4)	63.0	54 (6.1)
Store clerk	12 (3.5)	30.0	28 (5.3)	70.0	40 (4.6)
**Marital status**					
Married	108 (31.0)	38.3	174(32.8)	61.7	282 (67.9)
Single	240 (69.0)	40.3	356 (67.2)	59.7	596 (32.1)
**Gestational age**					
1st Trimester	97(27.9)	35.3	178 (33.6)	64.7	275 (31.3)
2nd Trimester	148 (42.5)	39.2	230 (43.4)	60.8	378 (43.1)
3rd Trimester	103 (29.6)	45.8	122 (23.0)	54.2	225 (25.6)
**Number of births**					
0	79 (22.7)	35.6	143(27.0)	64.4	222 (25.3)
1	79(22.7)	32.6	163 (30.8)	67.4	242 (27.6)
≥2	190 (54.6)	45.9	224 (42.2)	54.1	414 (47.1)
**Spontaneous abortion**					
Yes	27 (7.8)	60.0	18 (3.4)	40.0	45 (5.1)
No	321 (92.2)	38.5	512 (96.6)	61.5	833 (94.9)
**Hepatitis B infection**					
Negative	245 (70.4)	37.6	407 (76.8)	62.4	652 (74.3)
Positive	103 (29.6)	45.6	123 (23.2)	54.4	226 (25.7)
**HIV status**					
Negative	287 (82.5)	37.8	473 (89.2)	62.2	760 (86.6)
Positive	61 (17.5)	51.7	57 (10.8)	48.3	118 (13.4)
**Awareness of toxoplasmosis**					
Does not know anything about the disease	318 (91.4)	39.2	494 (93.2)	60.8	812 (92.5)
Heard speak, but do not know anything about it	26(7.5)	47.3	29 (5.5)	52.7	55 (6.3)
Know anything about the disease	4(1.1)	36.4	7 (1.3)	63.6	11 (1.2)
**Pre-natal consultation was performed in all pregnancies**					
Yes	335 (96.3)	39.2	519 (97.9)	60.8	854 (97.3)
No	13 (3.7)	54.2	11 (2.1)	45.8	24 (2.7)
**History of miscarriage**					
Yes	217 (62.4)	45.4	261 (49.2)	54.6	478 (54.4)
No	131 (37.6)	32.8	269 (50.8)	67.2	400 (45.6)
**Pet at home**					
Cats	91 (26.1)	49.7	92 (17.4)	50.3	183 (20.8)
Dogs	11 (3.2)	31.4	24 (4.5)	68.6	35 (4.0)
Dogs and cats	4 (1.2)	100.0	0 (0.0	000.0	4 (0.5)
No	242 (69.5)	36.9	414 (78.1)	63.1	656 (74.7)
**Cats´defecating habits**					
Inside home	1 (0.3)	16.7	5 (0.9)	83.3	6 (0.7)
In the vicinity of the house	10 (2,9)	47.6	11 (2.1)	52.4	21 (2.4)
Far from the house	6 (1.7)	50.0	6 (1.1)	50.0	12 (1.4)
No	331 (95.1)	39.5	508 (95.9)	60.5	839 (95.5)
**Cats´ feeding habits**					
Raw / uncooked meat	1 (0.3)	33.3	2 (0.4)	66.7	3 (0.3)
Cooked meat	15 (4.3)	50.0	15 (2.8)	50.0	30 (3.4)
Cat food	1 (0.3)	16.7	5 (0.9)	83.3	6 (0.7)
No	331 (95.1)	39.5	508 (95.9)	60.5	839 (95.6)
**Contact with other animals**					
Yes	101 (29.0)	36.6	175 (33.0)	63.4	276 (31.4)
No	247 (71.0)	41.0	355 (67.0)	59.0	602 (68.6)
**Rats near the house**					
Yes	278 (79.9)	38.9	437(82.5)	61.1	715 (81.4)
No	70 (20.1)	42.9	93 (17.5)	57.1	163 (18.6)
**Sand/soil contact**					
Yes	69 (19.8)	43.9	88 (16.6)	56.1	157 (17.9)
No	279 (80.2)	38.7	442 (83.4)	61.3	721 (82.1)
**Recent blood transfusion**					
Yes	3 (0.9)	42.9	4 (0.8)	57.1	7 (0.8)
No	345 (99.1)	39.6	526 (99.2)	60.4	871 (99.2)
**Recently had some sting with needle or sharp objects**					
Yes	9 (2.6)	36.0	16 (3.0)	64.0	25 (2.8)
No	339 (97.4)	39.7	514 (97.0)	60.3	853 (97.2)
**Consumption of washed fruit/vegetables**					
Always	259 (74.4)	40.7	377 (71.1)	59.3	636 (72.4)
Sometimes	89 (25.6)	36.8	153 (28. 9)	63.2	242 (27.6)
**Raw/uncooked meat consumption**					
Yes	81 (23.3)	3**7**.5	135 (25.5)	62.5	216 (24.6)
No	267 (76.7)	40.3	395 (74.5)	59.7	662 (75.4)
**Consumes raw or undercooked egg**					
Yes	122 (35.1)	36.5	212(40.0)	63.5	334 (38.0)
No	226 (64.9)	41.5	318 (60.0)	58.5	544 (62.0)
**Consumption of meat from hunting sources**					
Yes	99 (28.4)	40.4	146 (27.5)	59.6	245 (27.9)
No	249 (71.6)	39.3	384 (72.5)	60.7	633 (72.1)
**Breeding animals for home consumption**					
Yes	29 (8.3)	43.9	37 (7.0)	56.1	66 (7.5)
No	319 (91.7)	39.3	493 (93.0)	60.7	812 (92.5)
**Access to basic sanitation**					
Yes	227 (65.2)	40.7	331 (62.5)	59.3	558 (63.6)
No	121 (34.8)	37.8	199 (37.5)	62.2	320 (36.4)
**Total**	**348**	**-**	**530**	**-**	**878**

The ages of the pregnant women ranged from 15 to 47 years, with an average of 29.7±5.8 (Mean±SD; median = 30) (Table 3). The majority of participants were between 26 and 35 years of age (n = 504). Ninety-nine percent of participants (n = 869) were educated above the primary grade level, and a lesser number had no occupation outside home (22.9%). In relation to marital status, 596 pregnant women reported being single (67.9%) and 282 (32.1%) married (Table 3).

In relation to gestational age, 275 (31.2%) were in the first trimester of pregnancy, 378 (43.1%) in the second trimester and 225 (25.6%) in the third trimester. The mean gestational age was 19.8±9.4 weeks (Mean±SD; median = 16). The majority of the pregnant women had more than three children (414; 47.1%), with a mean birth rate of 1.2±0.8 (Mean±SD; median = 1.0).

No children were born prematurely although 45 (5.1%) women suffered spontaneous abortion while the present study was under way (Table 3). Additionally, 854 (97.3%) women stated that they were monitored for routine prenatal assessment in all their pregnancies and 478 (54.4%) reported history of miscarriage.

Regarding other infectious diseases, 226 (25.7%) were seropositive for hepatitis B and 118 (13.4%) for HIV virus. We also observed a high number of pregnant women (812; 92.5%) who were unaware of *T*. *gondii* disease.

In relation to pets, 222 (25.3%) pregnant women had animals at home: 183 (20.85%) with dogs, 35 (4%) with cats and 4 (0.45%) with both. The majority of cats defecated outside home and were fed with food scraps (cooked) (30; 3.4%). The majority of the participants (602; 68.6%) stated that they only had contact with their own pets and not with those of others. Reports of the presence of rodents in or nearby the house were high (715, 81.4%) and a lesser number of pregnant women had contact with soil or were engaged in gardening (157, 17.9%) (Table 3).

In relation to blood transfusions, only 7 (0.8%) pregnant women reported having recently had a blood transfusion, and 25 (2.8%) reported having recently been subjected to a needle / syringe prick or intervention (Table 3).

The type of food consumed, as well as the conditions under which it was consumed, was also of interest in the present study: 636 (72.6%) wash fruits and vegetables before consumption; 216 (24.6%) consume raw or undercooked meat; 334 (38.0%) consume raw or undercooked eggs, and 245 (27.9%) reported having eaten game meat. Only 66 (7.5%) raise animals for individual consumption. Over half of the women (558; 63.6%) answered that they have proper garbage disposal, drinking water and toilets at home.

As expected, Luanda had a greater number of *T*. *gondii* seropositive pregnant women (179 of 348 positive cases; 51.4%) since it has the larger population surveyed (435 of 878; 49.5%) (Table 4). The other municipalities had 168 positive cases (48.6%) and we did not have participants from Quissama. However, the prevalence of toxoplasmosis was similar in almost over municipalities: 43.8% in Cazenga (28 of 64); 42.5% in Viana (88 of 207); 42.3% in Cacuaco (22 of 52); and 41.1% in Luanda (179 of 435). In contrast, the seroprevalence in municipality of Belas was lower when compared to the other municipalities (25.8%; 31 of 120) (Table 4).

**Table 4:** Number of *Toxoplasma gondii* seropositive and seronegative pregnant women, by municipalities of the province of Luanda, Angola.

Municipality	Positive n (%)	Seroprevalence (%)	Negative n (%)	Seroprevalence (%)	Total
Belas	31 (9.0)	25.8	89 (16.8)	74.2	120 (13.7)
Cazenga	28 (8.0)	43.8	36 (6.8)	56.2	64 (7.3)
Cacuaco	22 (6.3)	42.3	30 (5.7)	57.7	52 (5.9)
Luanda	179 (51.4)	41.1	256 (48.3)	58.9	435 (49.5)
Viana	88 (25.3)	42.5	119 (22.4)	57.5	207 (23.6)
Icole and Bengo	-	-	-	-	-
Quissama	-	-	-	-	-
**Total**	**348**	**-**	**530**	**-**	**878**

In the univariate analysis, the risk factors associated with toxoplasmosis seroprevalence included the maternal age (p <0.0001), gestational age (3rd trimester, p = 0.0328), have children (p = 0.026), spontaneous abortion (p = 0.0048), hepatitis B (p = 0.040) and HIV seropositive (p = 0.005), history of miscarriage (p = 0.0001), and owning pets (dog, cat or both) (p = 0.032) (Table 5). Other analyzed factors such as education, employment, contact with sand/soil, consumption of fruit/vegetables and meat ingestion were not associated with seropositivity of the surveyed population.

**Table 5:** Univariate analysis of associated risk factors for seropositivity of IgG anti-*T*. *gondii* antibodies in 878 pregnant women, from 2016 to 2017, in Luanda province, Angola.

Variables	Positive	Total	OR (95%CI)	*p-*value
	n (%)	n (%)		
**Age range**				
≤ 25 years old	60 (25.8)	233(26,5)	0.4299 (0.3084; 0.5993)	< 0.0001*
> 25 years old	288 (44.7)	645 (73.5)		
**Education**				
Low (up to elementary school)	142 (40.6)	350 (39.9)	1.067 (0.809; 1.406)	0.6726
High (high school or higher education)	206 (39.0)	528 (60.1)		
**Employment**				
No (homemakers)	86 (42.8)	201 (22.9)	1.185 (0.8606; 1.630)	0.3246
Yes	262(38.7)	677(77.1)		
**Marital status**				
Married	108 (38.3)	282 (67.9)	0.9207 (0.6885; 1.231)	0.6053
Single	240 (40.3)	596 (32.1)		
**Gestational age**				
Pregnant woman (1st-2nd trimester)	245 (37.5)	653 (74.4)	0.7113 (0.5235; 0.9663)	0.0328*
Pregnant woman (3rd trimester)	103 (45.8)	225 (25.6)		
**Children**				
Yes	269(41.0)	656(74.7)	1,445 (1,051; 1,986)	0,0260*
No	79 (35.6)	222 (25.3)		
**Spontaneous abortion**				
Yes	27 (60.0)	45 (5.1)	2.393 (1.297;4.415)	0.0048*
No	321 (38.5)	833 (94.9)		
**Hepatitis B infection**				
Yes	103 (45.6)	226 (25.7)	1. 391 (1.024; 1.889)	0.0400*
No	245 (37. 6)	652 (74.3)		
**HIV status**				
Yes	61 (51.7)	118 (13.4)	1.764 (1.194; 2.605)	0.0050*
No	287 (37.8)	760 (86.6)		
**History of miscarriage**				
Yes	217 (45. 4)	478 (54.4)	1.707 (1.296; 2.249)	0.0001*
No	131 (32.8)	400 (45.6)		
**Pet at home**				
Yes	106 (47.7)	222 (25.3)	1.417 (1.041; 1.927)	0.0320*
No	242 (36.9)	656 (74.7)		
**Own cats**				
Yes	18 (46.2)	39 (4.4)	1.322 (0.694; 2.519)	0.4070
No	330 (39.3)	839 (95.6)		
**Contact with animals other than their own**				
Yes	101 (36.6)	276 (31.4)	0.830 (0.618; 1.113)	0.2350
No	247 (41.0)	602(68.6)		
**Rats near the house**				
Yes	278 (38.9)	715(81.4)	0.845 (0.599; 1.193)	0.3750
No	70 (43.0)	163 (18.6)		
**Sand/soil contact**				
Yes	69 (43.9)	157 (17.9)	1. 242 (0.876; 1.761)	0.2420
No	279 (38.7)	721 (82.1)		
**Recent blood transfusion**				
Yes	3 (42.9)	7 (0.8)	1.143 (0.254; 5.143)	1.0000
No	345 (39.6)	871 (99.2)		
**Recently had some sting with needle or sharp objects**				
Yes	9 (36.0)	25 (2.8)	0.8529 (0.3725; 1.953)	0.8366
No	339 (39.7)	853 (97.2)		
**Consumption of washed fruit/vegetables**				
Always	259 (40.7)	636 (72.4)	1.199 (0.883; 1. 629)	0.2790
Sometimes	89 (36.8)	242 (27.6)		
**Raw/uncooked meat consumption**				
Yes	81(37.5)	216 (24.6)	0.888 (0.647; 1.218)	0.4720
No	267 (40.3)	662 (75.4)		
**Consumes raw or undercooked egg**				
Yes	122 (36.5)	334 (38.0)	0.793 (0.599; 1.143)	0.1180
No	226 (41.5)	544(62.0)		
**Consumption of meat from hunting sources**				
Yes	99 (40.4)	245 (27.9)	1.046 (0.774; 1.413)	0.8180
No	249 (39.3)	633 (72.1)		
**Breeding animals for home consumption**				
Yes	29 (43.9)	66 (7.5)	1.211 (0.730; 2.010)	0.5130
No	319 (39.3)	812 (92.5)		
**Access to basic sanitation**				
Yes	227 (40.7)	558 (63.6)	1.128 (0.851; 1.496)	0.4310
No	121 (37.8)	320 (36.4)		
**Total**	**348**	**878**		

OR: Odds Ratio, CI: Confidence Interval * Statistically significant (p<0.05).

The bivariate logistic regression analysis has shown a significant increased risk associated to toxoplasmosis seroprevalence with pregnant women in the last trimester (OR = 1.521; p = 0.0225), suffering spontaneous abortion (OR = 2.404; p = 0.00499) and having pets (OR = 1.573; p = 0.00386) (Table 6). Regarding the presence of co-infections, pregnant women who tested seropositive for hepatitis B (OR = 1.400; p = 0.0311) or HIV (OR = 1.773; p = 0.00396) were also associated with an increased risk for toxoplasmosis. Also showing an increased risk for toxoplasmosis were pregnant women from 26–35 years of age (OR = 2.063; p = 0.000027) and from 36–47 years of age (OR = 2.258; p = 0.00029), and those having three or more children (OR = 1.631; p = 0.0245). The multivariate logistic regression analysis (adjusted by age) confirm a significant increased risk for toxoplasmosis in women in the last trimester of pregnancy (OR = 1.457; p = 0.0435), suffering spontaneous abortion (OR = 1.863; p = 0.0457), having pets at home (OR = 1.658; p = 0.0015), testing seropositive for hepatitis B (OR = 1.375; p = 0.0437) or HIV (OR = 1.833; p = 0.0027) (Table 6).

**Table 6:** Binomial logistic regression models for the final analysis of risk factors associated with seropositivity of IgG anti-*T*. *gondii* antibodies in 878 pregnant women samples, from 2016 to 2017, in Luanda province, Angola.

Variables	OR	95% CI	*p*-value	OR	95% CI	*p*-value
	*Unadjusted*			*Adjusted by age*		
**Maternal age** (Ref = ≤25 years)						
26–35	2.063	1.476–2.909	0.000027*	-		
36–47	2.258	1.455–3.518	0.00029*	-		
**Gestational age** (Ref = 1st trimester)						
2nd	1.180	0.856–1.632	0.312	1.165	0.841–1.616	0.3582
3rd	1.521	1.061–2.185	0.0225*	1.457	1.011–2.102	0.0435*
**Number of births** (Ref = 0)						
1 and 2	1.190	0.859–1.656	0.2987	1.005	0.715–1.417	0.9753
≥ 3	1.525	0.996–2.337	0.0054*	1.127	0.682–1.858	0.6390
**Spontaneous abortion** (Ref = No)						
Yes	1.984	1.086–3.667	0.0263*	1.863	1.014–3.465	0.0457*
**Hepatitis B infection** (Ref = Negative)						
Positive	1.400	1.030–1.901	0.0311*	1.375	1.008–1.874	0.0437*
**HIV status** (Ref = Negative)						
Positive	1.773	1.201–2.623	0.0039*	1.833	1.233–2.730	0.0027*
**History of miscarriage** (Ref = 0)						
≥1	1.726	1.311–2.278	0.00010*	1.320	0.945–1.849	0.1038
**Pet at home** (Ref = 0)						
≥1	1.573	1.156–2.140	0.00386*	1.658	1.212–2.269	0.0015*
**Residence** (Ref = Luanda)						
Belas	0.479	0.305–0.737	0.00104*	0.471	0.299–0.728	0.0009*
Cazenga	1.135	0.667–1.917	0.559	1.149	0.671–1.956	0.6079
Cacuaco	0.836	0.452–1.510	0.636	0.796	0.427–1.447	0.4616
Viana	1.074	0.767–1.502	0.673	1.084	0.770–1.522	0.6418

OR: Odds Ratio, CI: Confidence Interval * Statistically significant (p<0.05).

Potential associations between the area of residence and toxoplasmosis were also examined (Table 6). Pregnant women residing in the municipality of Belas were less likely to be seropositive for *T*. *gondii* by bivariate (OR 0.479; p = 0.00104) and by multivariate analysis (OR 0.471; p = 0.0009) compared with other municipalities.

Of the 878 pregnant women, the DNA was extracted from the placenta fragments of only two participants, who were positive for IgM and IgG antibodies. Corroborating with the results of the IgG avidity test (high values), indicating a chronic infection, both samples were negative for the *T*. *gondii* B1 locus gene (S4 File).

**Fig 3:**
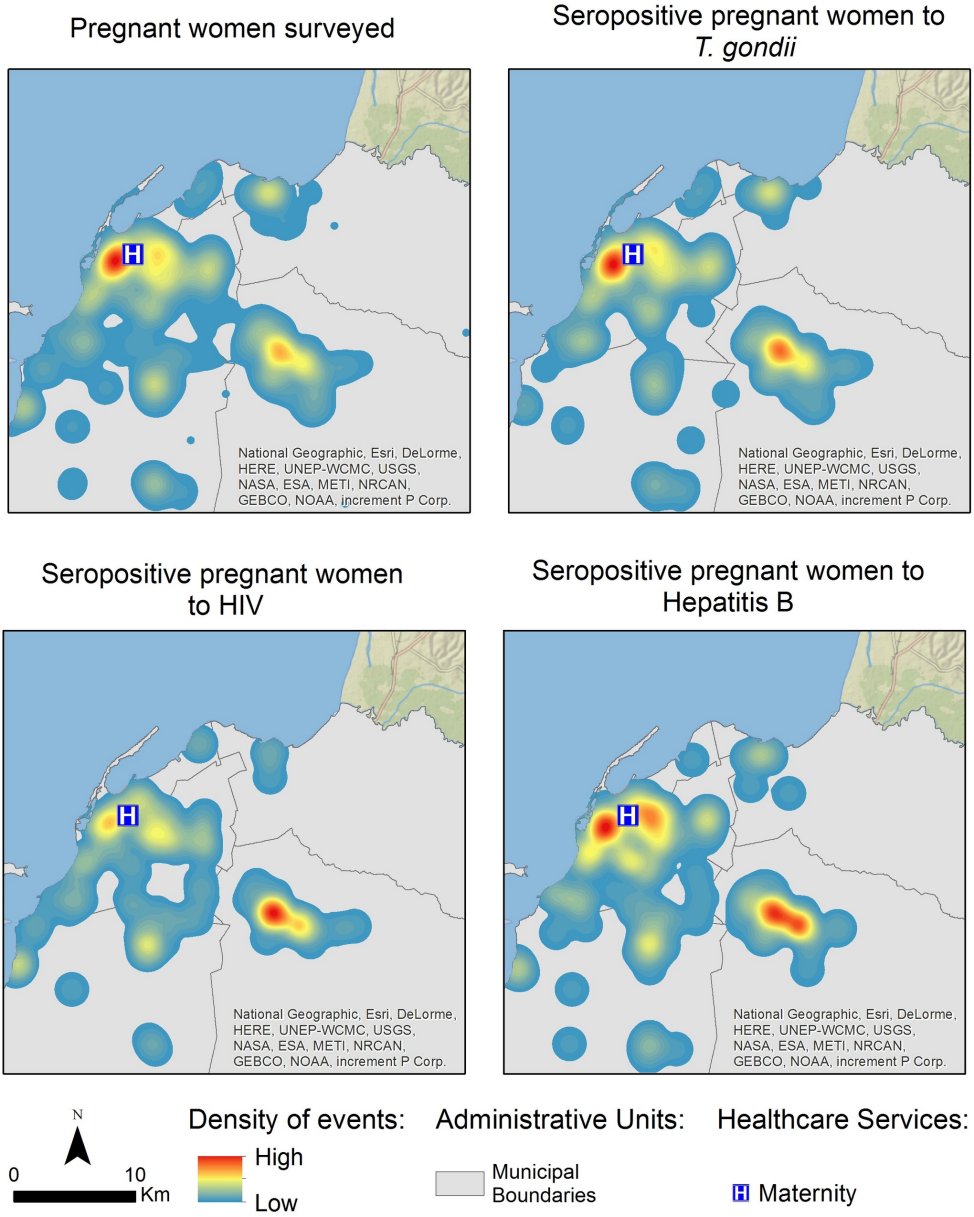
Geographical distribution and Gaussian kernel density surface map of pregnant women (A) with IgG anti-*T*. *gondii* antibodies (B), IgG anti-HIV antibodies (C) and IgG anti-Hepatitis B virus antibodies (D) in Luanda, Angola.

The geospatial distribution of pregnant women seropositive for *T*. *gondii*, HIV and hepatitis B antibodies in Luanda can be observed in Fig 3. Based on participants’ place of residence we observed a marked geographical pattern, with a high incidence density near Lucrecia Paim Maternity. The geographical distribution of pregnant women with and without antibodies to *T*. *gondii*, HIV and hepatitis B was similar, and we observed a statistically significant spatial dependency.

## Discussion

The current study estimated the prevalence of *T*. *gondii* infection among pregnant women from Luanda province (Angola), evaluating some epidemiological aspects and identifying the risk factors for toxoplasmosis in this population. Spatial analysis was applied for the first time to the case of Luanda to describe the geographical distribution of toxoplasmosis, HIV and Hepatitis B in pregnant women.

To measure seroprevalence of toxoplasmosis, anti-*T gondii* IgM and IgG quantification were performed on pregnant women attending the Lucrécia Paim Maternity Hospital (LPMH) from August 2016 to May 2017.

The seroprevalence of toxoplasmosis in our study was 39.6%, and this relatively high value has been consistently reported by several studies conducted in similar populations [20–23], even though the prevalence is higher when compared to a previous study performed in Luanda (27.3%) [16]. This difference might be related to the method used in the quantification of anti-*T*. *gondii* IgG and IgM antibodies (Electrochemiluminescence) which has a high sensitivity and specificity.

It is well documented that the prevalence of toxoplasmosis varies across countries, and indeed contrasting data are observed: 6.2% in México [24]; 4.5% to 5.8% in Vietnam [25]; 44.5% in Tanzania [23]; 80.3% in the Democratic Republic of Congo [26]; and 85.3% in Ethiopia [27]. The differences in prevalence rates in the worldwide population may be associated with various factors related to each region/country and specific characteristics of the population as well with the diagnostic methods used. Other examples include sanitary practices and eating/drinking habits, presence of household felines, poor or lower socioeconomic status, contact with soil, lack of knowledge about the disease, and the overall climate conditions that allow for the protracted environmental survival of the parasite’s oocysts. In addition, felids infected with *T*. *gondii* are largely asymptomatic and can spread the infection, contributing to high infection rates [12,28,29].

Previous studies report the relationship between maternal infection with congenital toxoplasmosis, which is relatively low (<20%) during the first trimester and increases to up to 90% at the end of gestation [10–11]. Recently, the global prevalence of acute *Toxoplasma* infection in pregnant women was estimated at about 1.1% [30]. This prevalence rate represents a significant burden of infection in pregnant women, and suggests that a large number of newborns are at risk of acquiring congenital toxoplasmosis *in utero*. In the present study, the seronegative pregnant women, which represented 60.4% of the participants, had follow-up during prenatal care and none of them acquired toxoplasmosis. Initially, there was a suspicion of active toxoplasmosis in two participants who tested positive for IgM and IgG; however, the IgG avidity test allowed the exclusion of recent infection in these two women’s. The IgG avidity test that evaluates the strength of binding of the IgG antibodies to *T*. *gondii* antigens, allowing the exclusion of a chronic infection (those of high avidity), without risk to the fetus. The use of IgM and IgG avidity tests, together with the analysis of gestational age, has beneficial results in terms of determining the risk of vertical transmission throughout the gestation, thus becoming a model for decision making that is inexpensive and that avoids research and unnecessary treatments, in some cases [31].

The characteristics of the individual pregnant women and details on home conditions and food habits were collected using a structured questionnaire. The prevalence of *T*. *gondii* infection increases with age and the bivariate logistic regression analysis showed that there was a statistical association between the age groups and the positivity for IgG anti-*T*. *gondii*, with a higher probability of prior exposure for those aged 25 and over. These results were similar to the findings of most surveys that included not only pregnant women, postpartum and of reproductive age, but also those in the general population [32,33] and could be explained by the increase in exposure to sources of infection throughout life [34,35].

The multivariate logistic regression analysis (adjusted to age) revealed that gestational age, actual spontaneous abortion and the presence of household pets increased the risk of *T*. *gondii* seropositivity. However, contact with sand/soil, the type of food consumed and the conditions under which food is consumed did not have any significant statistical correlation with *T*. *gondii* seropositivity in pregnant women.

The percentage of pregnant women with prior exposure in the third trimester was high compared with the other trimesters, showing that women in the last trimester of pregnancy had an increased likelihood of *T*. *gondii* seropositivity.

Moreover, spontaneous abortion was the most common form of pregnancy loss in our study, and is in line with other studies [20,36,37]. We observed a significant association between maternal seropositivity of anti-*T*. *gondii* antibodies and spontaneous abortion. The high percentage of miscarriages can be explained by the fact that when primary infection in pregnant woman or reactivation in immunocompromised pregnant women occurs, tachyzoites can colonize the placenta tissues during the dissemination process and from there obtain access to the fetus [12,13]. In early pregnancy, transplacental passage of tachyzoites is a rare event, but the consequences are serious and one of them is miscarriage. The immunological control of placental infection is probably a key event in the occurrence of congenital infection and miscarriages [38], but advances in understanding the pathophysiological process still need to be achieved [13]. Unfortunately, we do not have fetus material or placenta from those cases of spontaneous abortion to confirm the presence of *T*. *gondii*. In the future, the implementation of the diagnosis of maternal primary infection as well the appropriate diagnosis of congenital toxoplasmosis will be crucial.

Angola is among the countries of the Southern Africa region with the lowest HIV prevalence rate, estimated at 2.35% in the adult population aged between 15–49, with 2.6% in women and 1.3% in pregnant women aged 15–49 [39,40]. HIV transmission in Angola is predominantly heterosexual, in 79.2% of reported cases, and the vertical transmission accounts for about 6% and blood transfusion for 0.5% (via transfusion and use of contaminated objects).

In the present study, the seroprevalence of *T*. *gondii* in HIV-positive pregnant women was higher than those who tested HIV-negative, with the difference being statistically significant. Similar results were reported in Ghana [41] and in Lusaka, Zambia [42], where the prevalence of *T*. *gondii* infection among pregnant HIV-positive women was also high. In contrast, however, a study carried out in northern Nigeria [43] found that the relationship between these two groups was not statistically significant.

The severity and clinical significance of human toxoplasmosis varies with the host's immune status [41]. Therefore, HIV-infected patients may be at higher risk of developing toxoplasmosis disease, especially when the TCD4+ cell count falls below 100 cells/L of blood [34]. Toxoplasmosis is the most common opportunistic infection in HIV-seropositive immunocompromised hosts, where it predominantly occurs as a reactivation of endogenous infection [41].

A higher prevalence of *T*. *gondii* infection was also observed among Hepatitis B seropositive pregnant women than in seronegative women, and the statistical analysis showed that Hepatitis B-positive women are at high risk to have toxoplasmosis. It was reported that hepatitis B infection in pregnant women also leads to increased risk of chronicity of cirrhosis and / or hepatocellular carcinoma [44]. Because of the prevalence of infection among the population, it is extremely important to perform serological screening in the prenatal period in order to initiate early treatment, or even avoid vertical transmission [45]. To have HIV and/or hepatitis B can lead to a reactivation of toxoplasmosis and, in some cases, trigger the most serious clinical manifestations of these diseases [44].

It is believed that cats are the main carriers and transmitters of *T*. *gondii* infection to humans [46]. Several studies have observed high IgG seropositivity related to the presence of cats [28,47]. In the present study, the seroprevalence of *T*. *gondii* among pregnant women who had cats was higher than in pregnant women who did not have cats. However, in our study no significant association was found between the presences of cats in the household and *T*. *gondii* seropositivity. Similar results were shown by studies conducted in Burkina Faso [21], the UK [48], Brazil [49], Turkey [50] and Nigeria [51]. In contrast, surveys performed in the USA [52], Ethiopia [27,53], and Taiwan [54] showed that pregnant women in contact with infected cats will naturally be at greater risk of acquiring the infection. It is important to mention that the risk of contracting *T*. *gondii* infection does not result simply from contact with domestic cats only [55]. Occasional contact with cats or owning them may not necessarily be a risk factor, while frequent exposure to feline stools [56,57] and/or neglect of preventive measures, i.e. not washing hands or wearing gloves, may increase the risk of infection to an appreciable level [58]. On the other hand, having cats as household pets in Angola is not a common practice. However, street cats are usually seen, which can pose a serious risk to toxoplasmosis since they may be responsible for much of the environmental contamination with oocysts in the water or soil that may remain viable for years [59].

We found that having pets at home, not specifically the cat, is a potential risk factor for *T*. *gondii* seropositivity in pregnant women. The logistic regression analysis showed that contact with pets at home and/or in family member’s/friends’ homes is associated with *T*. *gondii*–seropositive women. Cats can disseminate oocysts in the environment, while dogs can act as mechanical vectors assisting in the transmission of infectious forms of *T*. *gondii* [60].

Basic sanitary conditions can contribute to the transmission of several infectious diseases. In Angola, according to data from Census 2014, only 47% of households have access to appropriate sources of drinking water: 46% using tankers and 28.9% using public water [17,18]. There is a contrast in water supply levels between urban and rural areas of the country. The urban population with access to drinking water is around 67%, while the rural population is only 32%. Between provinces differences in access to adequate drinking water are also seen. Moreover, about one third of households (32%) have some kind of appropriate and unshared sanitation facility, with the proportion higher in urban areas (46%) than in rural areas (11%). Although our data do not show an association between the basic sanitation conditions (specifically source of drinking water) and *T*. *gondii* seropositivity, we emphasized that most pregnant women were from urban areas with better sanitary conditions.

The distribution of *T*. *gondii* seropositive pregnant women varied among the municipalities that comprise Luanda province, with the highest number of cases in municipalities with a larger population: Luanda, Viana, Belas, Cazenga and Cacuaco. The location of the LPMH maternity hospital is determinant, given its location in the center of Luanda and its easy access from the other municipalities, reasons for the population to commonly resort to the referred maternity hospital. However, the seroprevalence and the risk to acquire toxoplasmosis in the municipality of Belas was lower when compared to the other municipalities.

Previous studies have shown that living in rural or suburban regions with soil exposure is a risk factor for toxoplasmosis in pregnant women [58,61]. Our results point that the prevalence of *T*. *gondii* among pregnant women is higher in the group in frequent contact with the soil; however, no statistically significant association was observed between *T*. *gondii* seropositivity and activities and practices that promote contact with soil (gardening). This result may have been influenced by the fact that most of the pregnant women live in urban area and do not have contact with soil.

One of the risk factors often associated with acute toxoplasmosis in pregnant women is eating raw or undercooked meat [62]. Our results showed that *T*. *gondii* seropositivity in pregnant women was not associated with the consumption of raw / undercooked meat or the consumption of raw vegetables and fruits. These findings are in agreement with the study carried out in China [58] and Ethiopia [27] but in contrast with other reported studies [63–66]. These differences may be due to frequency of consumption, type of consumed meat and prevalence of the parasite in the animals [67,68]. Despite our data, *T*. *gondii* infection in animals (i.e. pigs, cattle, sheep, chickens, and goats) in the province of Luanda may contribute to toxoplasmosis transmission. Large-scale studies should be performed to estimate the association between this potential risk factor for *T*. *gondii* infection in Angola, including toxoplasmosis surveys in animals and the water supply.

The study of toxoplasmosis is important in the planning of educational programs aimed at reducing the incidence of toxoplasmosis during pregnancy [35]. The best measures to prevent toxoplasmosis are those directed at primary prevention, basically characterized by public health and education programs recommended through campaigns, lectures, pamphlets and the guidance provided by health teams informing pregnant women to avoid contact with animals, potentially harmful contaminated materials, and avoid the consumption of raw or undercooked meat. The use of gloves when handling the soil is also emphasized. A reduction of 63% in the first gestational infection can be obtained following these recommendations during pregnancy [31]. Unfortunately, routine screening for *T*. *gondii* infection during pregnancy is not performed for most Angolan women, as the test is not mandatory in prenatal care and the associated costs are high. In addition, poor access to higher education and the lack of promotion of public health in general explain how a very high percentage of the population lacks knowledge of toxoplasmosis as seen in the present study.

## Conclusions

There is a significant number of pregnant women in Luanda who are not immunized for toxoplasmosis and thus at risk of acquiring the primary infection during pregnancy and consequently infecting the fetus (congenital toxoplasmosis). Gestational age, the presence of pets at home, HIV-seropositivity and hepatitis B-seropositivity were identified as risk factors for *T*. *gondii* infection. However, contact with cats and sand/soil, the type of food consumed and the conditions under which food consumption occurs were not statistically significant and may play a secondary role in these particular female populations.

Therefore, it is crucial to include education on toxoplasmosis prevention in prenatal counseling, to enable the diagnosis of maternal primary infection as well the appropriate diagnosis of congenital toxoplasmosis. Our results also underscore the need for diagnostic and clinical follow-up of other infectious diseases, including HIV and hepatitis B during pregnancy.

More studies should be performed to identify the risk factors for toxoplasmosis in Angola, including *T*. *gondii* surveys carried out in other groups, in animals and the water supply.

## Supporting information

S1 File Ethics Committee, República de Angola, Ministério da Saúde. (PDF)

S2 File Questionnaire/Questionário de Recrutamento. (PDF)

S3 File Programs and datasets used to create the maps. (PDF)

S4 File Amplification of *Toxoplasma gondii* B1 gene locus by nested-PCR. (PNG)
