# Supplementary material for: Serological prevalence of toxoplasmosis in pregnant women in Luanda (Angola): Geospatial distribution and its association with socio-demographic and clinical-obstetric determinants
Source: PLoS One. 2020 Nov 6;15(11):e0241908. doi: 10.1371/journal.pone.0241908 (PMC7647088; doi:10.1371/journal.pone.0241908)
Supplement: S1 File — (PDF) [file pone.0241908.s001.pdf]

REPÚBLICA DE ANGOLA
MINISTÉRIO DA SAÚDE

COMITÉ DE ÉTICA

Nº 30 2019

Sobre o protocolo de investigação intitulado «**Prevalência, Caracterização Serológica e Molecular da Toxoplasmose em Mulheres Gravidas: Incidência da Toxoplasmose Congénita em Algumas Maternidades de Luanda**», submetido a este Comité pela **Senhora Amelia João Alice Nkutxi Vueba,** Estudante de Doutoramento em Ciências Farmacêuticas/Especialização Microbiologia e Parasitologia da Faculdade de Farmácia da Universidade de Coimbra/Portugal.

A leitura e análise do protocolo em epigrafe, permitiu ao Comité constatar a pretensão em contribuir na actualização do conhecimento e informações referentes a esta doença.

O projecto enquadra-se na iniciativa e estratégia do Ministério da Saúde na melhoria da qualidade dos serviços prestados à população e até nas acções de prevenção e promoção da saúde dos angolanos. O Comité considera o protocolo do estudo «**Positivo**», porque a sua investigação irá definir e implementar programas de prevenção primaria nas gravidas, uma vez que é fundamental e importante para a prevenção da toxoplasmose congénita.

FEITO EM LUANDA, AOS 23 DE SETEMBRO DE 2019.

A COORDENADORA DO CE
DRA. JOANA FILIPA M. M. AFONSO
