# Supplementary material for: Serological prevalence of toxoplasmosis in pregnant women in Luanda (Angola): Geospatial distribution and its association with socio-demographic and clinical-obstetric determinants
Source: PLoS One. 2020 Nov 6;15(11):e0241908. doi: 10.1371/journal.pone.0241908 (PMC7647088; doi:10.1371/journal.pone.0241908)
Supplement: S3 File — (PDF) [file pone.0241908.s003.pdf]

S3 File - Programs and datasets used to create the maps

The maps were produces using ArcMap 10.x.

Vectorial (i) points representing the mothers and the hospital was collected by the authors with GPS; ii) polygons representing the administrative regions were collected by the authors at the Angola National Statistics) and raster (heat maps were produced by the authors, based on the point collected with GPS) data was used.

The basemap, or background map are provided by ESRI and its credits are presented in each one of the maps (as the owners of the basemap define it). The text presented is:

Fig 1) Service Layer Credits: Sources: Esri, HERE, DeLorme, TomTom, Intermap, increment P Corp., GEBCO, USGS, FAO, NPS, NRCAN, GeoBase, IGN, Kadaster NL, Ordnance Survey, Esri Japan, METI, Esri China (Hong Kong), swisstopo, MapmyIndia, © OpenStreetMap contributors, and the GIS User Community Sources: Esri, DeLorme, USGS, NPS Sources: Esri, USGS, NOAA

Fig 3) Service Layer Credits: Sources: Esri, HERE, DeLorme, TomTom, Intermap, increment P Corp., GEBCO, USGS, FAO, NPS, NRCAN, GeoBase, IGN, Kadaster NL, Ordnance Survey, Esri Japan, METI, Esri China (Hong Kong), swisstopo, MapmyIndia, © OpenStreetMap contributors, and the GIS User Community
