# Supplementary figures and images for: Serological prevalence of toxoplasmosis in pregnant women in Luanda (Angola): Geospatial distribution and its association with socio-demographic and clinical-obstetric determinants
Source: PLoS One. 2020 Nov 6;15(11):e0241908. doi: 10.1371/journal.pone.0241908 (PMC7647088; doi:10.1371/journal.pone.0241908)

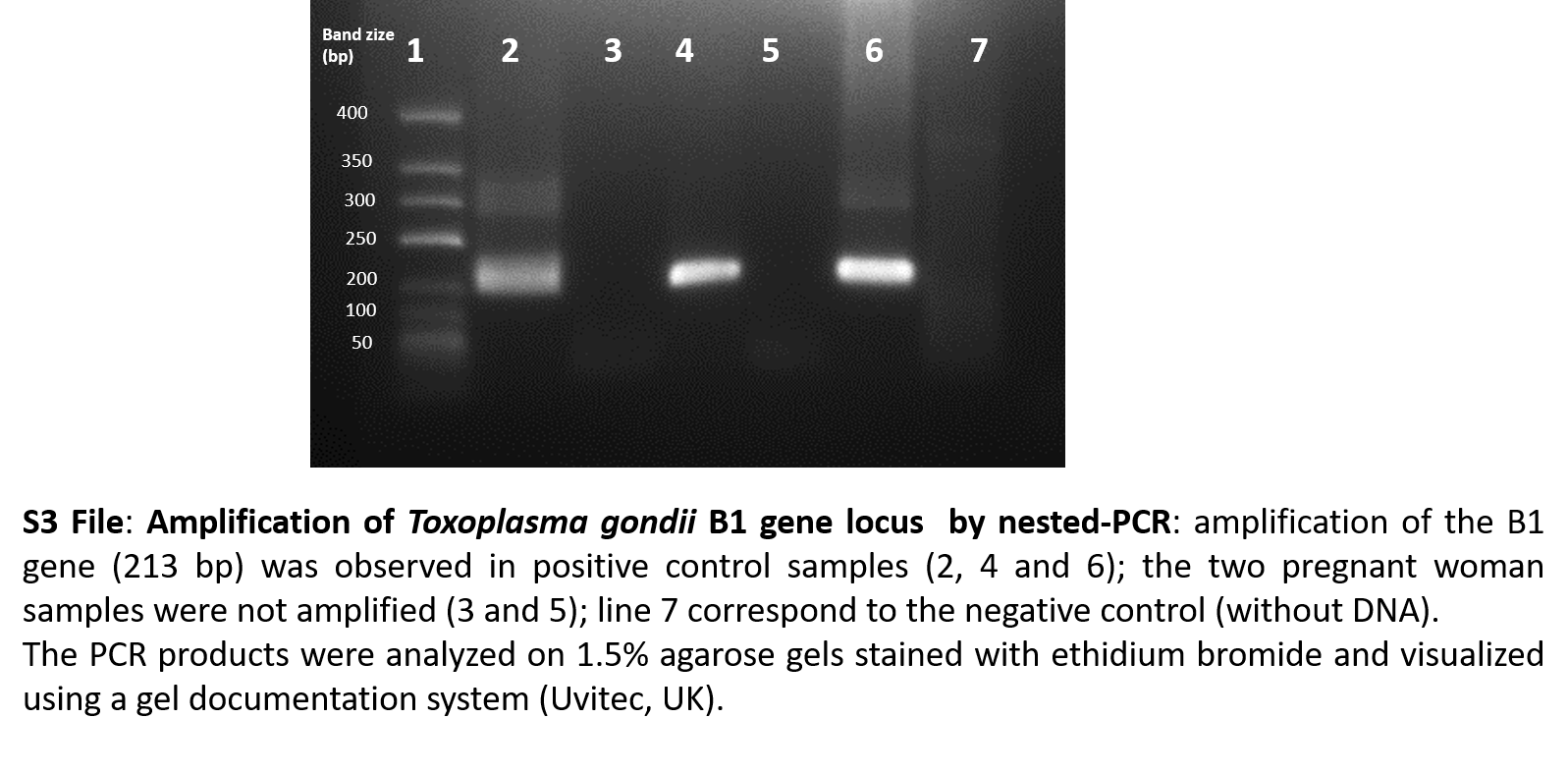

Supplement: S4 File — (PNG) [file pone.0241908.s004.png]
